# Young patients with colorectal cancer have poor survival in the first twenty months after operation and predictable survival in the medium and long-term: Analysis of survival and prognostic markers

**DOI:** 10.1186/1477-7819-8-82

**Published:** 2010-09-15

**Authors:** KK Chan, B Dassanayake, R Deen, RE Wickramarachchi, SK Kumarage, S Samita, KI Deen

**Affiliations:** 1 Consultant Colon and Rectal Surgeon, The Johor Bahru, Hospital, Johor, Malaysia; 2 The School of Biological Sciences, Cornell University, Ithaca, New York, USA; 3 The University Department of Surgery, North Colombo Teaching Hospital, Ragama, Sri Lanka; 4 The Department of Biostatistics, Postgraduate Institute of Agriculture, University of Peradeniya, Sri Lanka; 5 Department of Surgery University of Kelaniya Medical School, Sri Lanka

## Abstract

**Objectives:**

This study compares clinico-pathological features in young (<40 years) and older patients (>50 years) with colorectal cancer, survival in the young and the influence of pre-operative clinical and histological factors on survival.

**Materials and methods:**

A twelve year prospective database of colorectal cancer was analysed. Fifty-three young patients were compared with forty seven consecutive older patients over fifty years old. An analysis of survival was undertaken in young patients using Kaplan Meier graphs, non parametric methods, Cox's Proportional Hazard Ratios and Weibull Hazard models.

**Results:**

Young patients comprised 13.4 percent of 397 with colorectal cancer. Duration of symptoms and presentation in the young was similar to older patients (median, range; young patients; 6 months, 2 weeks to 2 years, older patients; 4 months, 4 weeks to 3 years, p > 0.05). In both groups, the majority presented without bowel obstruction (young - 81%, older - 94%). Cancer proximal to the splenic flexure was present more in young than in older patients. Synchronous cancers were found exclusively in the young. Mucinous tumours were seen in 16% of young and 4% of older patients (p < 0.05). Ninety four percent of young cancer deaths were within 20 months of operation. At median follow up of 50 months in the young, overall survival was 70% and disease free survival 66%. American Joint Committee on Cancer (AJCC) stage 4 and use of pre-operative chemoradiation in rectal cancer was associated with poor survival in the young.

**Conclusion:**

If patients, who are less than 40 years old with colorectal cancer, survive twenty months after operation, the prognosis improves and their survival becomes predictable.

## Introduction

Colorectal cancer is the commonest malignancy in the gastrointestinal tract and the fourth leading cause of cancer associated death in the world. In the United States, it has been estimated that 108,070 new cases of colon cancer and 40,740 rectal cancers, respectively, would have been diagnosed in 2008 and 49,960 would have died from colorectal cancer [1]. Compared with the West, colorectal cancer in South and South East Asia has been reported to occur with a greater frequency in young patients (usually <40 years old) [2], although, in recent years, a population based study in the United States has shown an increase in the incidence of colorectal cancer in the young [3].

In general, colorectal cancer is a disease of the middle aged and elderly, with the majority diagnosed after the age of 55 years [4]. Some 2-10% of all colorectal cancers have been reported in young patients [4,5]. In older patients, survival curves after operation for colorectal cancer, as reported in most studies [6-8], assumes a "step-ladder" form with most deaths reported in the first three years. In young patients with colorectal cancer, survival has been reported to be poor compared with older patients [4] because, in the young , colorectal cancer is diagnosed late [9], often in advanced stage, and is cancer with poorer differentiation [10]. Reports of survival in young and older patients with colorectal cancer tend to differ [4,9,10].

The aim of this study was to compare clinical and pathological features of colorectal cancer in those less than 40 years old with patients older than 50 years, and, to specifically assess survival and factors that may influence survival in young patients having operation for colorectal cancer.

## Materials and methods

From September 1996 to September 2008, all patients less than 40 years old, diagnosed with colorectal cancer and treated at the department of surgery of the university of Kelaniya medical school, were analyzed from a prospective database. Systematic consecutive sampling was employed and all patients fulfilling the above requirements were included in the study. Selected patients' previous medical records, discharge summaries and histopathology reports were reviewed. Demographic data, presenting symptoms and their duration, pathological features of the tumour, tumour localization, histological data, pre-operative carcinoembryonic antigen (CEA) level, treatment modalities and survival data were scrutinized. The histology report of each patient was reviewed to determine histological subtypes [11], differentiation and stage of tumour. Tumours were staged according to the American Joint Committee on Cancer (AJCC) TNM staging system, 6^th ^edition. Histological types of tumour such as carcinoid, gastrointestinal stromal and neuroendocrine tumours were excluded.

Right sided lesions were classified as tumours proximal to splenic flexure whereas left sided lesions were classified as tumours from splenic flexure to the rectosigmoid junction. The rest were classified as rectal lesions. Synchronous tumours were defined as colon and rectal tumours detected either at pre-operative colonoscopy, at operation, or within 6 months of operation. All operative specimens were evaluated by a histopathologist. On examination of the histopathology specimen, an R0 resection refers to tumour margins which were free of microscopic tumour and, an R1 resection, where one or more margins of the histopathology specimen was observed to contain microscopic tumour.

Follow up was by direct communication with patients and their relatives in the out-patient clinic or by telephone or mail. The patient or a family member was contacted and interviewed to obtain further information. Patients were considered lost to follow up if the patient had failed to present at an out-patient clinic, or could not be contacted by telephone or letter, after more than a year. During follow up, patients were evaluated by history and physical examination, including digital rectal examination and CEA level every three months. A chest radiograph and trans-abdominal ultrasound scan or computerized tomogram was undertaken at one year. Annual colonoscopy was performed for the first two years and three years later, if individuals were found free of polyps or recurrent disease. Suspicious recurrent lesions were further evaluated with endoscopic ultrasound, computerized tomography, magnetic resonance imaging and positron emission tomography if appropriate. Clinical and pathological data of young patients with colorectal cancer were compared with forty seven patients from the same database, over 50 years old, in whom all comparable data were available (Table 1). Again, consecutive sampling as employed to avoid selection bias in the older age group, with the added inclusion criteria being age above 50 years and presence of complete records in all fields used for comparison. Furthermore, in younger patients, we evaluated overall survival after a diagnosis was made of colorectal cancer and significant prognostic markers of survival identified by first univariate and then multifactorial analysis. Disease free survival was analysed for all patients and also with reference to resection margin positivity (R0/1 status). Statistical analysis was performed using the *χ*^2 ^test or *t*-test as appropriate and survival probability was calculated using the Kaplan-Meier method. Prognostic factors for survival in young patients were analysed, using the Cox proportional hazards ratio, in univariate and multifactorial analyses. The two issues considered in fitting multifactorial models were whether the overall model was adequate, and if each factor was individually significant or redundant. This was undertaken since a statistical method was needed that adjusts for the effect of each factor such as R0/R1 status when assessing the effect of other factors. Hence Type III analysis using the Weibull Hazard model was used in factor assessment. Adequacy of model fit was established using the log likelihood ratio. Finally, hazard ratios were re-calculated using the multifactorial approach for factors found significant in type III analysis. All analyses were completed using the SAS System V 9.00, 2003 (SAS Institute, Cary, North Carolina, USA). *P *< 0.05 was considered significant.

**Table 1:** Comparison of young (n = 53) versus older (n = 47) patients with colorectal cancer

Variable	<40 years	>50 years	P value
	**Number (percent)**	**Number(percent)**	

**Gender**			
Male	26 (49%)	26 (55%)	0.53*
Female	27 (51%)	21 (45%)	

Duration of symptoms (Months)	7.9	6.6	0.44*

**Neo-Adjuvant Therapy**			
Received	8 (15%)	11 (23.4%)	0.29*
Not received	45 (85%)	36 (76.6%)	

Right hemicolectomy	6 (11%)	3 (6%)	

Left hemicolectomy	3 (6%)	1 (2%)	

Sigmoid colectomy	2 (4%)	4 (9%)	

Anterior resection	22 (41.5%)	30 (64%)	

Abdomino-perineal resection	4 (7.5%)	5 (11%)	

Subtotal colectomy	5 (9.4%)	Nil	

Hartmann's procedure	3 (5.7%)	3 (6%)	

Restorative proctocolectomy	8 (15.1%)	Nil	

Trans-anal excision	Nil	1 (2%)	

*Chi square test/t-test.

## Results

### Demography

From September 1996 to September 2008, 397 patients were treated for colorectal cancer. Fifty-three patients (13.4%) were young (mean age - 31.8 years; median 33 years and range -16 to 40 years). Gender ratio of young patients was almost equal. Comparison of clinical and pathological features in the young with older patients with colorectal cancer (median 66 years, range -50 to 89 years) did not show a difference in gender distribution, duration of symptoms and in the proportion of patients with rectal cancer having pre-operative chemoradiation (neo-adjuvant therapy) (Table 1). The median time of follow up for the young patients was 50 months (interquartile range 6 - 78 months).

### Clinical Presentation

The duration of symptoms at presentation in young patients was not different from older patients (young - 2 weeks to 2 years; mean 7.9 months and median 6 months compared with older patients - 4 weeks to 3 years; mean 6.6 months and median 4 months, Student's t-test, p > 0.05). The longest duration for a particular symptom was considered in determining duration of symptoms. In the young, the most common presenting symptom was alteration in bowel habit (47; 89%). Other symptoms were rectal bleeding (68%), non-specific abdominal pain (38%), tenesmus (24.5%), anaemia (6%) and loss of appetite or weight (9%). The majority were non-obstructing lesions with only ten patients (19%) presenting with acute and/or sub-acute intestinal obstruction. Likewise, in older patients, alteration in bowel habit was the commonest symptom at presentation (41, 87%) followed by rectal bleeding (85%), tenesmus (26%), non-specific abdominal pain (17%), loss of weight or appetite (17%) and anaemia (2%) respectively. Four patients (8.5%) of the older group presented with bowel obstruction. In most, symptoms were multiple.

### Treatment

Operation was performed in all with curative intent (Table 1). Eleven young patients (21%) had neo-adjuvant treatment before surgery for rectal cancer and 30 (57%) patients received post-operative adjuvant therapy overall. In the older group, eleven (23%) with rectal cancer received neo-adjuvant therapy and sixteen (34%) received post-operative adjuvant therapy.

### Pathological Characteristics of Tumour

The summary of tumour characteristics is shown in Table 2. Rectal cancer comprised the majority in the young and in older patients. In these young patients with fifty three index cancers and four synchronous cancers, forty eight (84%) tumours were adenocarcinoma without mucin, 5 (9%) were mucinous adenocarcinomas and 4 (7%) were of the signet ring variety. The majority of tumours were moderately differentiated. In older patients, forty three (92%) had adenocarcinoma without mucin, 2 (4%) had mucinous cancer and 2 (4%) had a signet cell cancer. Most cancers in older patients were moderately differentiated.

**Table 2:** Comparison of clinical and pathological features in young (n = 53) versus older patients (n = 47) having colorectal cancer

Variable		<40 years	≥50 years
Gender	Male	26 (51%)	26(45%)
	
	Female	27 (49%)	21(55%)

Duration of symptoms*	≤ 3 months	22 (41.5%)	22 (47%)

	>3 months	31 (58.5%)	25 (53%)

Tumour Location	Right colon (included 2 synchronous lesions in <40 year group)	10 (17.5%)	3 (6%)
	
	Left colon (included 2 synchronous lesions in <40 year group)	10 (17.5%)	13(28%)
	
	Rectal	37 (65%)	31 (66%)

Histological types	Adenocarcinoma (included 4 synchronous lesions in the young)	48 (84%)	43 (92%)
	
	Mucinous	5 (9%)	2 (4%)
	
	Signet ring	4 (7.0%)	2 (4%)

Tumor grade	Well	10 (18.9%)	2 (4%)
	
	Moderate	33 (62.2%)	43(92%)
	
	Poor	10 (18.9%)	2 (4%)

T stage	T0/1	5 (9.2%)	2 (4%)
	
	T2	11 (20.4%)	9 (19%)
	
	T3	24 (44.4%)	28 (60%)
	
	T4	11 (20.4%)	8 (17%)
	
	No residual tumor after chemoradiation	3 (5.6%)	Nil

N stage	N0	28 (51.8%)	25(54.3%)
	
	N1	13 (24.1%)	8 (17.4%)
	
	N2	13 (24.1%)	13 (28.3%)

AJCC staging *	I	7 (14.0%)	6 (12.8%)
	
	II	16 (32.0%)	20 (42.6%)
	
	III	24 (48.0%)	15 (31.9%)
	
	IV	3 (6.0%)	6 (12.8%)

Pre-operative CEA level (ng/ml)	<5.0	17 (51.5%)	16 (34%)
	
	>5.0	16 (48.5%)	31(66%)

Resection margin #	R0	40 (80.0%)	35 (74.5%)
	
	R1	10 (20.0%)	12 (25.5%)

*Data were not available in one of the older group. **# **Data were not available in three young patients.

Of 53 young patients, pre-operative CEA was available in 33 (mean CEA level - 20.8 ng/ml). A CEA level more than 5 ng/ml was considered abnormal. Seventeen patients (51.5%) had normal levels of CEA and 16 (48.5%) had abnormal pre-operative CEA levels. In older patients, mean CEA level was 37.8 ng/ml; 34% <5 ng/ml and 66% had a CEA level ≥5 ng/ml.

Histopathology staging of tumours in young patients (*n *= 50) revealed 23 (46%) with stage I/II disease, 24(48%) in Stage III and in the remaining 3(6%), liver metastasis or peritoneal deposits of tumour (stage IV) were diagnosed during operation. The majority of young patients (40 patients; 80%) had R0 resection. In the older age group, 55% were found to have stage I/II disease, 32% stage III and 13% with stage IV cancer. Like in young patients, the majority of older patients (75%) had R0 resection.

### Survival Analysis in the Young

**Figure 1:**
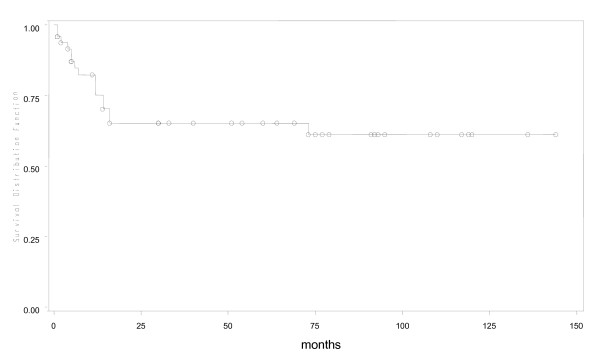
**Overall survival in young patients with colorectal cancer**.

**Figure 2:**
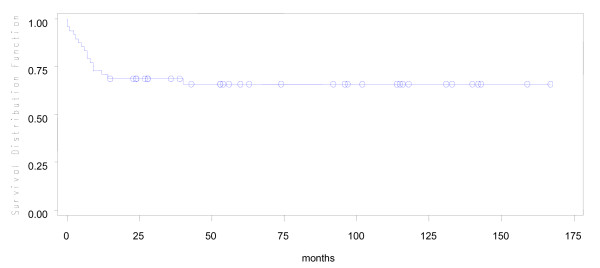
**Disease-free survival in young patients with colorectal cancer**.

**Figure 3:**
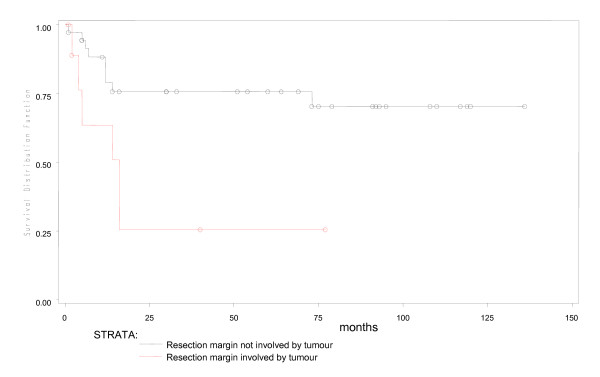
**Involvement of the resection margin by microscopic tumour and overall survival**.

**Figure 4:**
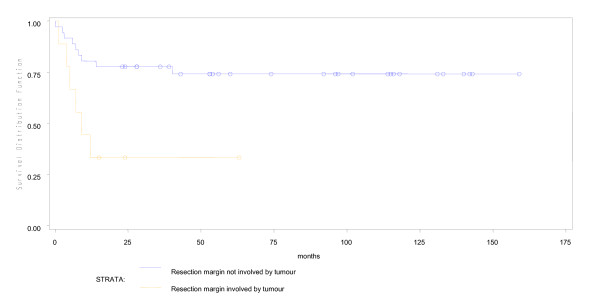
**Involvement of the resection margin by microscopic tumour and disease-free survival**.

During a median fifty months of follow-up in 53 young patients, 4 were lost to follow up early. Of the remaining 49, 16(30%) had expired. Predicted five year overall survival was 70% and disease free survival was 66% (Figures 1 and 2). Fifteen of 16 young patient deaths had occurred within 20 months of diagnosis. Eleven had died within the first year after surgery and 4 more in the following year. Only one patient had died after the second year. Fourteen (87.5%) of sixteen had died due to disseminated cancer and 2(12.5%) due to complications of adjuvant therapy. In a subset analysis of AJCC stage, 2 of 7(28.6%) with stage I cancer; 2 of 14(14.3%) with stage II; 6 of 22(27.3%) with stage III and all stage IV cancer patients had died. Most significantly, those who survived longer than 20 months were likely to live five or more years (Figure 1). In R0 patients, five-year overall survival was 79.3% (Figure 3), while five-year disease free survival was 74.2% (Figure 4).

**Figure 5:**
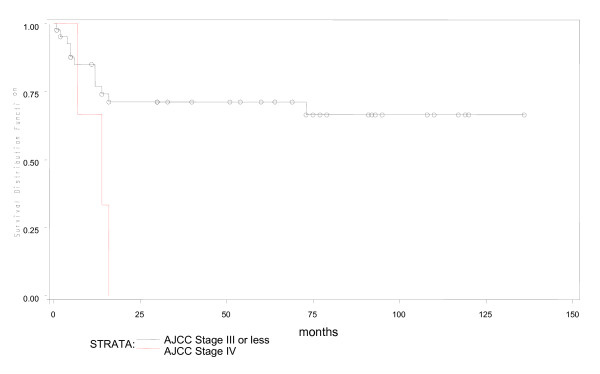
**AJCC stage I, II and III versus stage IV on overall survival**.

**Figure 6:**
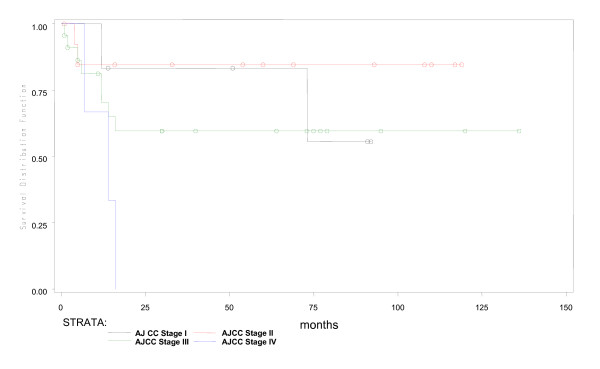
**Overall survival by AJCC stage**.

**Figure 7:**
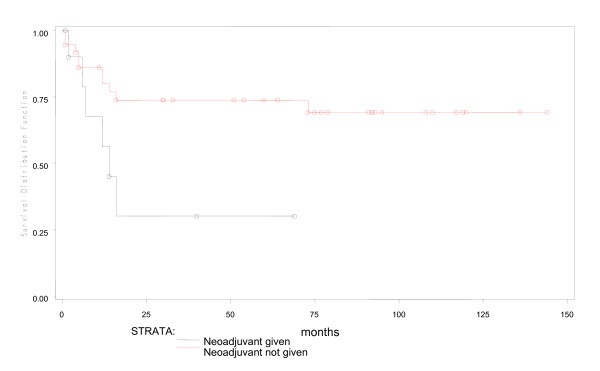
**Effect of neoadjuvant therapy for rectal cancer on overall survival**.

For overall survival, univariate analysis using Cox Proportional Hazard Model revealed that AJCC stage IV, a resection margin which was positive for tumour (i.e.R1) and the use of neo-adjuvant chemoradiation for rectal cancer was significantly associated with poor survival in young patients (Table 3, Figures 3, 5,6 and 7). Of 48 young patients with rectal cancer, eleven received neoadjuvant chemoradiation. Provision of neoadjuvant chemoradiation seemed a significant prognostic marker (p = 0.038, Cox proportional hazard ratio-3.01). Also, a disease-free resection margin (R0) appeared to have significant survival benefit compared to those with an R1 margin (Table 3). Both mucinous (5, 9.4%) and signet ring (4, 7.5%) tumours, each, had a mortality rate of 40% and 50% respectively compared to adenocarcinoma without mucin, 28% (p = 0.01). This was not significant in survival analysis however. Univariate analysis of other prognostic factors for overall survival did not show statistical significance (Table 3).

**Table 3:** Univariate analysis of prognostic factors in young patients - Cox Proportional Hazard Model

Variable	Factor Division	n	Hazard Ratio	p
Gender	Male	49	1.436	0.4751
				
	Female			

Neoadjuvant therapy	Not given	48	3.012	**0.0382**
				
	Given			

Duration of symptoms	≤ 3 months	39	0.804	0.7472
				
	>3 months			

CEA level	normal	33	1.508	0.5916
				
	elevated			

Tumour location	Right colon	43	1.021	0.9586
				
	Left colon			
				
	Rectum			

Histology	adenocarcinoma	46	1.237	0.7806
				
	mucinous			

Differentiation (1)	Well	46	1.716	0.2131
				
	Moderately			
				
	Poorly			

Differentiation (2)	Well	46	1.413	0.6510
				
	Non-well			

Differentiation (3)	Poorly	46	0.451	0.1475
				
	Non-poorly			

AJCC stage (1)	I	46	1.835	0.0799
				
	II			
				
	III			
				
	IV			

AJCC stage (2)	I	46	1.257	0.7637
				
	I1, 111 and 1V			

AJCC stage (3)	1 and II	46	2.524	0.1134

	II,111 and 1V			

AJCC stage (4)	1,11 and III	46	3.925	**0.0367**
				
	IV			

Resection Margin	R0	46	3.684	**0.0142**
				
	R1			

Perineural invasion	-	38	2.591	0.2304
				
	+			

Lymphatic invasion	-	37	2.909	0.1729
				
	+			

Vascular invasion	-	38	2.404	0.1460
				
	+			

Tumor margin	Pushing	13	0.817	0.8691
				
	Infiltrating			

Adjuvant therapy (1)	Given	39	3.350	0.2471
				
	Not Given			

Adjuvant therapy (2)	Chemotherapy	39	0.4174	0.6576

It is essential to note that both R0 and R1 patients were included in the study. R0 tumour resection represents an important prognostic factor for most malignant tumours. Therefore, to avoid bias, either R0 and R1 groups would have to be analysed separately, or a statistical method employed that adjusts for the effect of each factor such as R0/R1 status when assessing the effect of other factors. Thus, a Weibull Hazard model was fitted to evaluate the significance of neoadjuvant therapy, positive resection margins and AJCC stage 1V versus stage 1, 11 and 111. (The lifereg procedure, SAS 9.00): the model was first fitted using the three main effects only. Type III analysis of effects showed neo-adjuvant therapy (p = 0.014) and AJCC Stage IV versus stage III or less (p= 0.022) to be significant, while a positive resection margin (p = 0.1052) was not found to be significant. (Table 4)

**Table 4:** Multifactorial analysis - Weibull Hazard Model for Main effects

Type III Analysis of Effects		Log Likelihood
**Factor**	**p**	
Neo-adjuvant therapy (D)*	0.0142	
	
Resection Margin positivity (R)*	0.1052	-45.303
	
AJCC Stage IV vs. III or less (S)*	0.0225 *	

*D, R and S are assigned symbols used to assess interaction between factors in table Table 5

When 2-way interactions between the factors were added - first one at a time and then all three factors together - the improvement of model fit (log likelihood ratio) in all cases was also not significant when compared to the main effect model (Table 5). Based on results of Table 2, 3-way interaction was not studied, and the model with only the main effects assumed as adequate (Table 4). Therefore it was concluded that the two factors which were independently significant were neo-adjuvant therapy and AJCC Stage IV vs. III or less.

**Table 5:** Multifactorial analysis - Weibull Hazard Models for main effects and 2-way interactions. (Likelihood Ratios are calculated in comparison with the main effects model in Table 5)

Model	Type III Analysis of Effects		Log Likelihood	p (Likelihood Ratio)
		
1	Factors	p		
	
	Neoadjuvant therapy (D)	0.0062	-44.301	0.1568
			
	Resection Margin positivity (R)	0.0324		
			
	AJCC Stage IV vs. III or less (S)	0.2256		
			
	D*R	0.1649		
**2**	Neoadjuvant therapy (D)	0.0569	-45.294	0.8933
			
	Resection Margin positivity (R)	0.5757		
			
	AJCC Stage IV vs. III or less (S)	0.1435		
			
	R*S	0.8890		

**3**	Neoadjuvant therapy (D)	0.6231	-45.294	0.8933
			
	Resection Margin positivity (R)	0.1646		
			
	AJCC Stage IV vs. III or less (S)	0.1423		
			
	D*S	0.8890		

**4**	Neoadjuvant therapy (D)	0.0955	-44.232	0.5434
			
	Resection Margin positivity (R)	0.0974		
			
	AJCC Stage IV vs. III or less (S)	0.1856		
			
	D*R	0.1522		
			
	R*S	-		
			
	S*D	-		

Finally, multifactorial analysis using Cox Proportional Hazard Model of these two independently significant prognostic markers for survival in young patients was undertaken (AJCC Stage 1V vs. AJCC stage 111 or less and the use of neo-adjuvant therapy for rectal cancer). Hazard Ratios were calculated for these two factors. Both factors significantly affected survival in this model, neoadjuvant therapy showing a Hazard Ratio of 3.390 and AJCC Stage 1V vs. stage 111 or less a ratio of 4.009 with p < 0.05 in both cases (Table 6).

**Table 6:** Multifactorial analysis of significant prognostic factors in young patients by Cox Proportional Hazard Model (n = 45)

Factors	Hazard Ratio	p
**AJCC III or less vs. IV**	**4.009**	**0.0362**

**Neoadjuvant therapy**	**3.390**	**0.0265**

## Discussion

Over twelve years, fifty three of 397 (13.4%) patients treated at our centre comprised the young colorectal cancer group. Prevalence was comparable with most other reports from Asia; 10.1% in Taiwan [12], Istanbul 18% [13], another Sri Lankan report of 19.7% [14], and 23% in Saudi Arabia [15]. Our figure was considerably more than that reported from the West; 2.8% in the United States [9], 3% in France [9] and 5.5% in New Zealand [6,15]. The high percentage reported in developing countries may be due, in part, to the higher population of younger people in these countries.

Young patients with colorectal cancer may be diagnosed late due to low suspicion of malignancy in these patients [9]. However, the duration of presentation did not seem to influence overall survival in this analysis and we are in agreement with Lin et al [16]. The most common presenting symptom was alteration of bowel habit. Other symptoms were rectal bleeding, non-specific abdominal pain, tenesmus, anaemia, loss of appetite and weight. The majority were not obstructive lesions. Furthermore, in this study, the majority of young patient cancers were sporadic with a greater frequency in the colon compared with older patients. Synchronous cancers were to be found exclusively in young patients. Hence, young patients of Asian origin, who present with these symptoms, should be investigated without delay to exclude malignancy.

Our study showed that a majority of young patients had adenocarcinoma without a mucinous component and, that about one in 5 were poorly differentiated, which was greater than in older patients. Mucinous and signet ring cell cancer comprised 16% of all colorectal cancers in the young. This differs from most other reports where mucinous, signet ring and poorly differentiated tumour comprised the majority of pathology [4,10,12,17]. One study had both mucinous and signet ring cancer as the leading type [15]. In general, mucinous and signet ring tumours have been associated with higher mortality compared with carcinoma without a mucin component.

In the young, predicted survival at five years was 70% and disease free survival was 66%. Our findings are in accordance with several previous reports which also includes a previous report by our group for survival in older patients with colorectal cancer [8,12,13] , where Kaplan Meier graphs for older patients have already been displayed and discussed [8]. Unique, in this study, is that death from cancer in those less than 40 years occurred early, within twenty months of operation, which is different to cancer related death reported in those over 50 years in our previous report [8]. In other words, those young patients who survived more than 20 months after operation were likely to live five years and more. Our data are different to previous reports, in which, overall five year survival rates, in young patients with colorectal cancer, were around 30% [10,18]. Greater five year survival in our patients may be due to the smaller proportion of mucinous and signet ring tumours compared with a higher prevalence of mucin producing, high grade tumours reported in other studies.

Earlier AJCC stage and non-use of neo-adjuvant therapy in patients with rectal cancer seemed to bear significant survival benefit. The association between use of neo-adjuvant therapy for rectal cancer and poor survival may reflect aggressive tumour biology and later tumour stage rather than the beneficial effect of pre-operative chemoradiation on rectal cancer. Furthermore, although univariate analysis showed a positive resection margin to be associated with poor survival, The Weibull Hazard model analysis did not find this to be a significant independent prognostic factor. We may infer that a positive resection margin in colorectal cancer, given that the surgical procedure was performed with curative intent by a trained surgeon, was a summative co-factor in a biologically aggressive tumour.

In our analysis, other variables such as gender, tumour location, tumour characteristics - invasion margin (pushing vs. infiltrative), perineural and lymphovascular invasion - did not significantly influence overall survival. Limitations in the current study may be attributed to a small sample size in a single institution.

## Conclusion

We found that mortality in young patients with colorectal cancer was greatest in the first 20 months after operation. Contrary to some previous reports, survival beyond twenty months after operation in young patients improves and is predictable.

Prognostic markers for survival were stage of disease and the use of pre-operative chemo-radiation for rectal cancer.

## Competing interests

The authors declare that they have no competing interests.

## Authors' contributions

KKC - Tabulated data, wrote the manuscript in draft. BED -Undertook all of the statistical analysis and contributed to several drafts of the paper. RID - Helped in data collection and tabulation and provision of data for survival analysis. RW - Collection of data, wrote the manuscript in draft. SKK -Contributed to drafts of the manuscript.

SS - Supervised and assisted in all of the statistical analysis. KID - Conceived of the idea, participated in its design and wrote and supervised several drafts of the manuscript. All authors have read and approve of the final manuscript.

## References

[B1] Jemal A Siegel R Ward E Hao YP Xu JQ Murray T Thun M J. Cancer Statistics, 2008 CA: A Cancer Journal for Clinicians 2008 58 71 96 10.3322/CA.2007.0010 18287387

[B2] Chew MH Koh PK Ng KH Eu KW Improved survival in an Asian cohort of young colorectal cancer patients: an analysis of 523 patients from a single institution Int J Colorectal Dis 2009 24 9 1075 83 10.1007/s00384-009-0701-7 19387661

[B3] O'Connell JB Maggard MA Liu JH Etzioni DA Livingston EH Ko CY Rates of Colon and Rectal Cancers are Increasing in The Young The American Surgeon 2003 69 866 872 14570365

[B4] O'Connell JB Maggard MA Liu JH Etzioni DA Livingston EH Ko CY Do Young Colon Cancer Patients Have Worse Outcome? World Journal of Surgery 2004 28 558 562 10.1007/s00268-004-7306-7 15366745

[B5] Lim GCC Halimah Y2nd Report of the National Cancer Registry: Cancer Incidence in Malaysia 2003 2004 Kuala Lumpur, National Cancer registry

[B6] Keating J Yong D Cutler G Johnstone J Multidisciplinary treatment of colorectal cancer in New Zealand: survival rate from 1987 to 2002 NZ Med J 2006 119 U2238 16998579

[B7] Andreoni B Chiappa A Bertani E Bellomi M Orecchia R Zampino M Fazio N Venturino M Orsi F Sonzogni A Pace U Monfardini L Surgical outcomes of colon and rectal cancer over a decade: results from a consecutive monocentric experience in 902 unselected patients World J Surg Oncol 2007 5 73 10.1186/1477-7819-5-73 17610720 PMC1959229

[B8] Perera T Wijesuriya RE Suraweera PHR Wijewardene K Kumarage SK Ariyaratne MHJ Deen KI The prevalence of colorectal cancer and survival in patients from the Gampaha District, North Colombo region The Ceylon Medical Journal 2008 53 17 21 18590265 10.4038/cmj.v53i1.221

[B9] Adloff M Arnaud JP Schloegel M Thibaud D Bergarmaschi R Colorectal Cancer in Patients Under 40 Years of Age Diseases of the Colon & Rectum 1986 29 322 325 10.1007/BF02554121 3009108

[B10] Smith C Butler JA Colorectal Cancer in Patients Younger Than 40 Years of Age Diseases of the Colon & Rectum 1989 32 843 846 10.1007/BF02554552 2791769

[B11] Hamilton SR Aaltonen LA Pathology and Genetics of Tumours of the Digestive System 2000 Lyon, France: International Agency for Research on Cancer

[B12] Chen HS Curative Resection of Colorectal Adenocarcinoma: Multivariate Analysis of 5-Year Follow-up World Journal of Surgery 1999 23 1301 1306 10.1007/s002689900666 10552125

[B13] Alici S Aykan Faruk N Sakar B Bulutlar G Kaytan E Topuz E Colorectal cancer in Young Pateints: Characteristics and Outcome Tohoku J. Exp. Med 2003 199 85 93 10.1620/tjem.199.85 12705353

[B14] De Silva MV Fernando MS Fernando D Comparison of Some Clinical and Histological Features of Colorectal Carcinoma Occurring in Patients Below and Above 40 years Ceylon Medical Journal 2000 45 166 168 11293963 10.4038/cmj.v45i4.6722

[B15] Isbister WH Colorectal cancer Below Age 40 in The Kingdom of Saudi Arabia Australian and New Zealand Journal of Surgery 1992 62 468 472 10.1111/j.1445-2197.1992.tb07227.x 1590715

[B16] Lin JT Wang WS Yen CC Liu JH Yang MH Chao TC Chen M Chiou TJ Outcome of Colorectal Carcinoma in Patients under 40 years of age Journal of Gastroenterology and Hepatology 2005 20 900 905 10.1111/j.1440-1746.2005.03893.x 15946138

[B17] Liang JT Huang KC Cheng AL Jeng YM Wu MS Wang SM Clinicopathological and molecular biological features of colorectal cancer in patients less than 40 years of age British Journal of Surgery 2003 90 205 214 10.1002/bjs.4015 12555297

[B18] Domergue J Ismail M Astre C Saint-Aubert B Joyeux H Solassol C Pujol H Colorectal carcinoma in patients younger than 40 years of age. Montpellier Cancer Institute experience with 78 patients Cancer 1988 61 835 840 10.1002/1097-0142(19880215)61:4<835::AID-CNCR2820610432>3.0.CO;2-X 3338041

